# ﻿A new species of *Hemiphyllodactylus* (Squamata, Gekkonidae) from southwest Yunnan, China

**DOI:** 10.3897/zookeys.1197.117359

**Published:** 2024-04-17

**Authors:** Hongxin Zhou, Shimin Li, Ziqi Shen, Shuo Liu, Dingqi Rao

**Affiliations:** 1 Key Laboratory for Forest Resources Conservation and Utilization in the Southwest Mountains of China, Ministry of Education Faculty of Biodiversity and Conservation, Southwest Forestry University, Kunming, Yunnan 650224, China Kunming Institute of Zoology, Chinese Academy of Sciences Kunming China; 2 Kunming Institute of Zoology, Chinese Academy of Sciences, Kunming, Yunnan 650201, China Southwest Forestry University Kunming China; 3 Anhui Normal University, Wuhu, Anhui 241000, China Anhui Normal University Wuhu China; 4 Kunming Natural History Museum of Zoology, Kunming Institute of Zoology, Chinese Academy of Sciences, Kunming, Yunnan 650223, China Kunming Natural History Museum of Zoology, Chinese Academy of Science Kunming China

**Keywords:** Gengma Dai and Wa Autonomous County, *Hemiphyllodactylusgengmaensis* sp. nov., integrative taxonomy, molecular phylogeny, slender gecko

## Abstract

A new species of gekkonid, *Hemiphyllodactylusgengmaensis***sp. nov.**, is described based on six specimens from Gengma Dai and Wa Autonomous County, Yunnan, China. The new species can be distinguished from its congeners by a significant genetic divergence of greater than 9.7% in the mitochondrial ND2 gene and a combination of the following characters: a maximum SVL of 43.24mm; 8 or 9 chin scales; six circumnasal scales; 2 or 3 internasal scales; 9–11 supralabial scales; 8 or 9 infralabial scales; 11–18 dorsal scales; 8–10 ventral scales; a manual lamellar formula of 5–5–5–4 or 5–6–5–4 and a pedal lamellar formula of 5–5–6–5; 20–25 precloacal and femoral pore-bearing scales contiguous in males; dark postorbital stripes or striping on body; dark dorsal transverse blotches present; and a brown postsacral mark bearing anteriorly projecting arms. The discovery of this new species brings the number of *Hemiphyllodactylus* species in China to 15.

## ﻿Introduction

Species of the genus *Hemiphyllodactylus* Bleeker, 1860 are small nocturnal geckos (SVL < 63 mm) distributed in South Asia, Southeast Asia, South China, and the Indo-Pacific region (Zug 2010; Grismer et al. 2013, 2018a; Agarwal et al. 2019; Eliades et al. 2019; Agung et al. 2021, 2022). Furthermore, all *Hemiphyllodactylus* are well camouflaged, occur in low densities, are forest-dwelling, and have small populations (Zug 2010; Grismer et al. 2013, 2018a; Agarwal et al. 2019; Eliades et al. 2019; Agung et al. 2021, 2022). Hence, *Hemiphyllodactylus* was considered to a low-diversity taxon (Zug 2010) until Grismer et al. (2013) revealed its high diversity using integrative taxonomy. In the following decade, the number of species within the group has increased from 12 to 54 (Uetz et al. 2023).

In China, *Hemiphyllodactylus* species have also been overlooked. The first species of slender geckos known from China is *Gehyrayunnanensis* (Boulenger, 1903), then “Smith revised its taxonomic status, placing it in the genus *Hemiphyllodactylus*” (Zug 2010). Zhou et al. (1981) recognized three subspecies of *H.yunnanensis* based on the digital lamellae patterns of specimens collected in Yunnan, Guizhou, and Guangxi Zhuang Autonomous Region, China: *H.y.dushanensis* Zhou & Liu, *H.y.jinpingensis* Zhou & Liu, and *H.y.longlingensis* Zhou & Liu. However, for the subsequent 32 years, most of Chinese slender geckos were regarded as simply *H.yunnanensis*, until Grismer et al. (2013) elevated these three subspecies to full species rank. Subsequently, nine additional species were incrementally recorded from China: *H.zugi* Nguyen, Lehmann, Le Duc, Duong, Bonkowski & Ziegler, 2013; *H.changningensis* Guo, Zhou, Yan & Li, 2015; *H.huishuiensis* Yan, Lin, Guo, Li & Zhou, 2016; *H.hongkongensis* Sung, Lee, Ng, Zhang & Yang, 2018; *H.zayuensis* Jiang, Wang & Che, 2020; *H.dupanglingensis* Zhang, Qian & Yang, 2020; *H.zhutangxiangensis* Agung, Grismer, Grismer, Quah, Chornelia, Lu & Hughes, 2021; *H.simaoensis* Agung, Chornelia, Grismer, Grismer, Quah, Lu, Tomlinson & Hughes, 2022; and *H.yanshanensis* Agung, Chornelia, Grismer, Grismer, Quah, Lu, Tomlinson & Hughes, 2022. The fourteenth species, *H.typus* Bleeker, 1860, as a widely distributed species, its actual coordinate data for distribution in China is unavailable (Agung et al. 2021; Uetz et al. 2023). According to recent genus-wide molecular phylogenetic studies, all *Hemiphyllodactylus* species in China belong to the *typus* group, and they are divided into four clades (Grismer et al. 2013, 2014a, 2014b, 2017, 2018a, 2020a, 2020b; Ngo et al. 2014; Agung et al. 2021, 2022): both clade 3 (*H.longlingensis*, *H.zhutangxiangensis*, *H.zayuensis*, and *H.changningensis*) and clade 4 (*H.jinpingensis* and *H.simaoensis*) of Agung et al. (2022) colonized China from western Indochina, and both clade 6 (*H.dushanensis*, *H.hongkongensis*, *H.dupanglingensis*, *H.zugi*, *H.huishuiensis*, and *H.yanshanensis*) and clade 7 (*H.yunnanensis*) of Agung et al. (2022) colonized China from eastern Indochina.

During our herpetological survey in Banxing Village, Gengma Dai and Wa Autonomous County, Yunnan, China, we collected six specimens belonging to the genus *Hemiphyllodactylus*. These specimens are distinguished from known species of *Hemiphyllodactylus* based on molecular and morphological data. Therefore, we describe them as a new species below.

## ﻿Materials and methods

### ﻿Sampling

**Figure 1.**
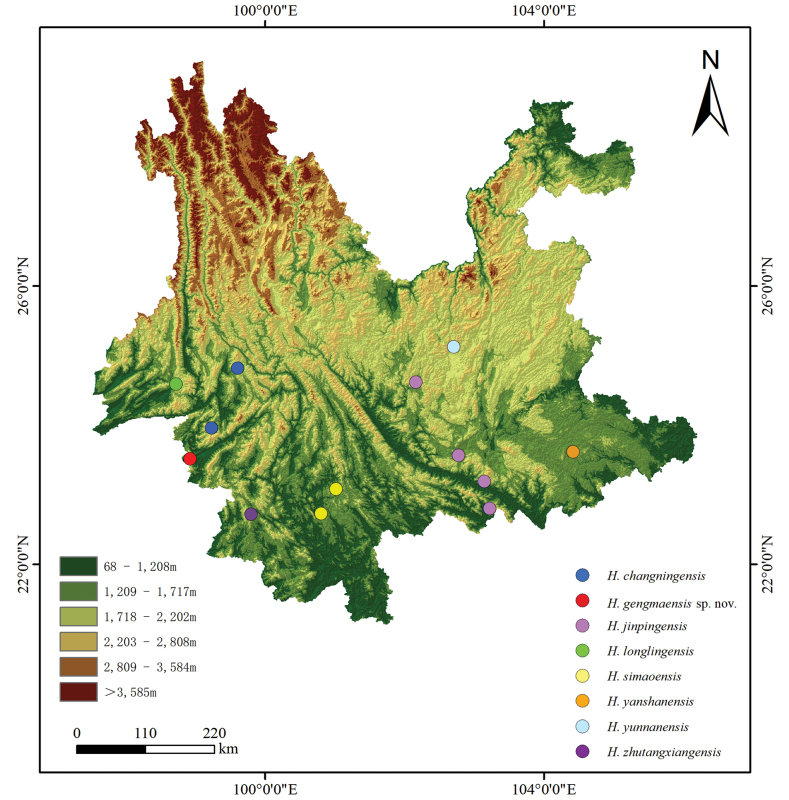
Distribution map of the genus *Hemiphyllodactylus* in Yunnan Province, China.

Six specimens were collected from in Banxing Village, Gengma Dai and Wa Autonomous County of Yunnan Province in China on 15 May 2014 (Fig. 1). All specimens were preserved in 80% ethanol, and their muscle and liver tissues were preserved in 95% ethanol. Specimens were deposited in Kunming Institute of Zoology (**KIZ**), Chinese Academy of Sciences (**CAS**).

### ﻿Molecular data and phylogenetic analyses

We used Trelief Hi-Pure Animal Genomic DNA Kit for genomic DNA extraction following the manufacturer’s protocol (https://www.tsingke.com.cn). We amplified and sequenced the complete mitochondrial NADH dehydrogenase subunit 2 gene (ND2), totaling 1,038 bp useing the primers L4437b and H5934 (Macey et al. 1997). The protocol for polymerase chain reaction (PCR) amplifications followed Agung et al. (2021). Genomic DNA extraction, PCR processes, and sequencing were executed at Beijing Tsingke Biotechnology Co., Ltd. All specimen sequences have been deposited in GenBank, with accession numbers from PP540021 to PP540025 .

A total of 47 ND2 sequences from GenBank, containing 2 ND2 sequences of outgroup taxa (*Hemiphyllodactylusharterti* Werner, 1900) and 45 sequences of extant *Hemiphyllodactylus* species, was downloaded; these with our five new sequences are listed in Table 1. Sequences were assembled and manual proofread in SeqMan (DNASTAR, Inc., Madison, WI, USA), then aligned using Clustal W (Thompson et al. 1994) implemented in MEGA 7 (Kumar et al. 2016). For phylogenetic relationships analysis, we considered maximum likelihood (ML) and Bayesian inference (BI) using IQ-TREE v. 2.2.0 (Nguyen et al. 2015) and MrBayes v. 3.2.7a (Ronquist et al. 2012) in the Phylosuite application (Zhang D et al. 2020; Xiang et al. 2023), respectively. After alignment, we used Gblock 0.91b (Talavera and Castresana 2007) to remove misaligned positions. ModelFinder v. 2.2.0 (Kalyaanamoorthy et al. 2017) was used to select the best-fitting model of evolution based on the Bayesian Information Criterion (BIC). A maximum-likelihood (ML) analysis was conducted using TPM+F+G4 as the best-fit substitution model for codon position one, TPM+F+G4 for position two, and TIM+F+G4 for position three. We applied 1,000 bootstrap pseudoreplicates with the ultrafast bootstrap approximation algorithm (UFBoot) (Agung et al. 2021), where nodes having values 95 and above were considered highly supported (Minh et al. 2013). A Bayesian-inference (BI) analysis was conducted using GTR+I+G+F model following the methods by Agung et al. (2021), except that instead of discarding 10% of the trees, we discarded the first 25% of the sampled as burn-in. Nodes with Bayesian posterior probabilities (BPP) of 0.95 and above were considered highly supported (Huelsenbeck et al. 2001; Wilcox et al. 2002). Uncorrected pairwise divergences were calculated using MEGA 7 (Kumar et al. 2016).

**Table 1.** List of specimens used for phylogenetic analyses in this study.

Species	GenBank no.	Locality	Voucher information
*H.harterti*	KF219760	Bukit Larut, Malaysia	LSUHC 10383
*H.harterti*	KF219761	Bukit Larut, Malaysia	LSUHC 10384
*H.indosobrinus*	JN393935	Champasak, Pakxong, Laos	FMNH 258695
*H.flaviventris*	MG322161	Chanthaburi, Thailand	ZMKU TM001204N
*H.flaviventris*	MG322162	Chanthaburi, Thailand	ZMKU TM001205N
*H.flaviventris*	MG322163	Chanthaburi, Thailand	ZMKU TM001206N
*H.flaviventris*	MG322164	Chanthaburi, Thailand	ZMKU TM001207N
*H.flaviventris*	MG322165	Chanthaburi, Thailand	ZMKU TM001208N
*H.arakuensis*	MK570109	Araku, Visakhapatnam District, Andhra Pradesh, India	BNHS 2275
*H.aurantiacus*	MK570110	Yercaud, Salem District, Tamil Nadu, India	AK 237
*H.aurantiacus*	MK570111	Yercaud, Salem District, Tamil Nadu, India	AMB s.n.
*H.jnana*	MK570112	Bangalore, Karnataka, India	CES G174
*H.jnana*	MK570113	Bangalore, Karnataka, India	CES G173
*H.jnana*	MK570114	Bangalore, Karnataka, India	CYL01
*H.jnana*	MK570115	Bangalore, Karnataka, India	CES G470
*H.kolliensis*	MK570116	Kolli Hills, Namakkal, Tamil Nadu, India	CES G138
*H.kolliensis*	MK570117	Kolli Hills, Namakkal, Tamil Nadu, India	AK 276
*H.zwegabinensis*	MT028174	Zwegabin Mountain, Kayin State, Myanmar	LSUHC 14184
*H.pinlaungensis*	MT028166	Pinlaung City, Shan State, Myanmar	LSUHC 14263
*H.pinlaungensis*	MT028167	Pinlaung City, Shan State, Myanmar	LSUHC 14264
*H.pinlaungensis*	MT028168	Pinlaung City, Shan State, Myanmar	LSUHC 14265
*H.kyaiktiyoensis*	MT028146	Mon State, Myanmar	LSUHC 14030
*H.kyaiktiyoensis*	MT028147	Mon State, Myanmar	LSUHC 14031
*H.kyaiktiyoensis*	MT028148	Mon State, Myanmar	LSUHC 14032
*H.kyaiktiyoensis*	MT028149	Mon State, Myanmar	LSUHC 14033
*H.khlonglanensis*	MG322153	Kamphaeng Phet, Thailand	ZMKU TM000999N
*H.khlonglanensis*	MG322154	Kamphaeng Phet, Thailand	ZMKU TM001000N
*H.khlonglanensis*	MG322155	Kamphaeng Phet, Thailand	ZMKU TM001001N
*H.khlonglanensis*	MG322156	Kamphaeng Phet, Thailand	ZMKU TM001002N
*H.zhutangxiangensis*	MW962150	Zhutangxiang town, Lancang Lahu, Yunnan, China	KIZ061163
*H.zhutangxiangensis*	MW962151	Zhutangxiang town, Lancang Lahu, Yunnan, China	KIZ061164
*H.zhutangxiangensis*	MW962152	Zhutangxiang town, Lancang Lahu, Yunnan, China	KIZ061165
*H.zhutangxiangensis*	MW962153	Zhutangxiang town, Lancang Lahu, Yunnan, China	KIZ061166
*H.zhutangxiangensis*	MW962154	Zhutangxiang town, Lancang Lahu, Yunnan, China	KIZ061167
*H.longlingensis*	FJ971045	Longyang District, Baoshan, Yunnan, China	isolate N30
*H.longlingensis*	FJ971046	Longyang District, Baoshan, Yunnan, China	NJNUh00104
*H.longlingensis*	FJ971047	Longyang District, Baoshan, Yunnan, China	isolate N32
*H.longlingensis*	FJ971048	Longyang District, Baoshan, Yunnan, China	isolate N33
*H.zalonicus*	MW039150	Zalon Taung National Forest, Ban Mauk, Sagaing, Myanmar	ZMMU R 16635
*H.changningensis*	ON676073	Yongde County, Yunnan, China	KIZ 061990
*H.changningensis*	ON676074	Yongde County, Yunnan, China	KIZ 061991
*H.changningensis*	ON676075	Yongde County, Yunnan, China	KIZ 061992
*H.changningensis*	ON676076	Yongde County, Yunnan, China	KIZ 061993
*H.changningensis*	ON676077	Yongde County, Yunnan, China	KIZ 061994
*H.changningensis*	ON676078	Yongde County, Yunnan, China	KIZ 061995
*H.changningensis*	ON676079	Yongde County, Yunnan, China	KIZ 061996
*H.changningensis*	ON676080	Yongde County, Yunnan, China	KIZ 061997
*H.gengmaensis* sp. nov.	PP540023	Gengma Dai and Wa Autonomous County, Yunnan, China	2014002297
*H.gengmaensis* sp. nov.	PP540024	Gengma Dai and Wa Autonomous County, Yunnan, China	2014002298
*H.gengmaensis* sp. nov.	PP540022	Gengma Dai and Wa Autonomous County, Yunnan, China	2014002299
*H.gengmaensis* sp. nov.	PP540021	Gengma Dai and Wa Autonomous County, Yunnan, China	2014002300
*H.gengmaensis* sp. nov.	PP540025	Gengma Dai and Wa Autonomous County, Yunnan, China	2014002302

### ﻿Morphological data

Mensural data were taken with a digital calipers to the nearest 0.01 mm under a dissecting microscope (Jiangnan XTB–01) following Zug (2010), Grismer et al. (2013), and Agung et al. (2021): snout–vent length (**SVL**) taken from the tip of the snout to the vent ; tail length (**TL**) taken from the vent to the tip of the tail ; trunk length (**TrunkL**) taken from the posterior margin of the forelimb at its insertion point on the body to the anterior margin of the hind limb at its insertion point on the body ; head length (**HL**) measured from the posterior margin of the retroarticular process of the lower jaw to the tip of the snout ; head width (**HW**) measured at the angle of the jaws ; eye diameter (**ED**) the greatest horizontal diameter of the eyeball ; snout–eye length (**SnEye**) measured from anterior-most margin of the eyeball to the tip of snout ; nares–eye length (**NarEye**) measured from the anterior margin of the eyeball to the posterior margin of the external nares ; and snout width (**SnW**) measured between the external nares.

For meristic characters and color pattern, we measured and evaluated them according to the methods of Agung et al. (2021).

## ﻿Results

**Figure 2.**
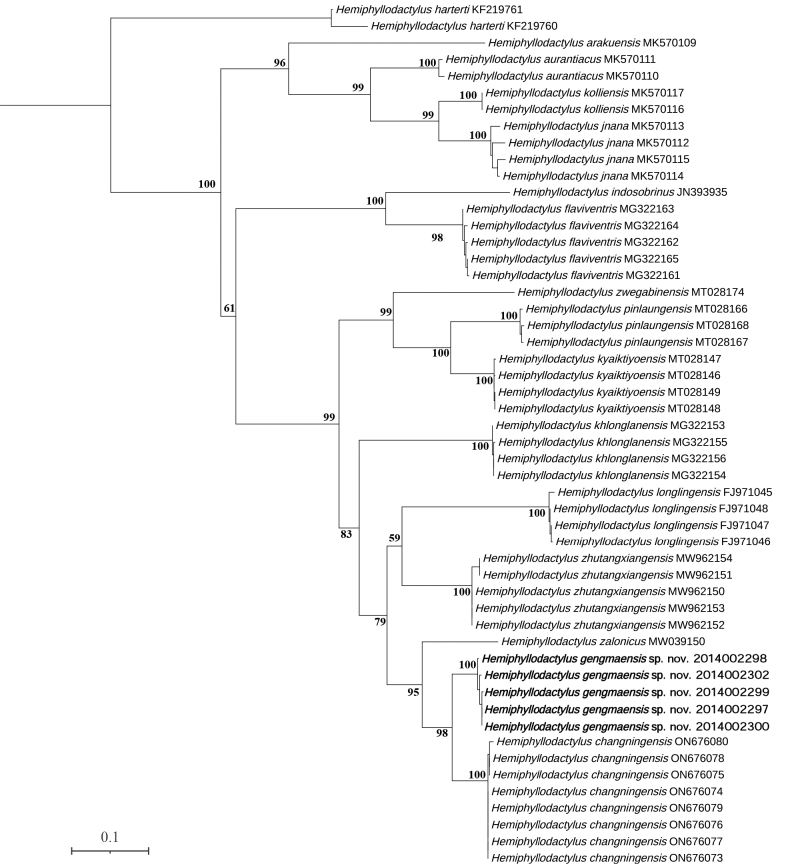
Maximum-likelihood consensus tree based on 1038 bp mitochondrial ND2 gene. Numbers by the nodes indicate ML bootstrap support values.

**Figure 3.**
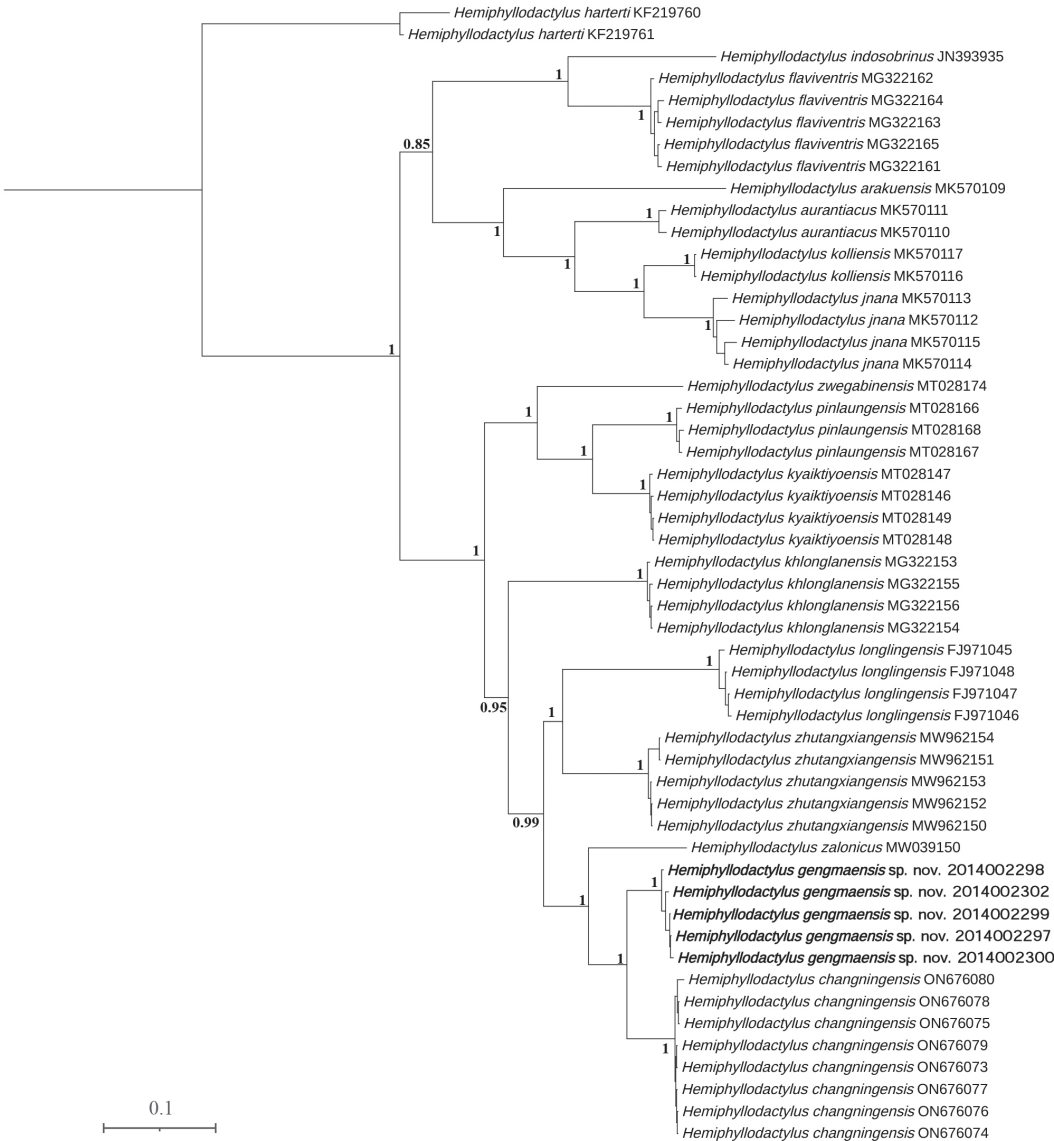
Phylogenetic tree by Bayesian inference based on 1038 bp mitochondrial ND2 gene. Numbers by the nodes indicate posterior probability values of the BI.

Our results of ML and BI analyses were similar to those obtained by Agung et al. (2021): the specimens from Gengma County were recovered as members of Clade 3 in both ML and BI analyses (Figs 2, 3), which includes *Hemiphyllodactyluslonglingensis*, *H.zalonicus*, *H.changningensis*, and *H.zhutangxiangensis*. The mean percentage of uncorrected pairwise distance between the Gengma County specimens and *H.changningensis* is 9.7% (Table 2). Furthermore, the new species also could be distinguished from its congeners by body proportions, CN, VS, Lamellar formulae hands and feet II–V, SL1T and total number of femoroprecloacal pores. Therefore, we describe them here as a new species.

**Table 2.** The mean percentage of the uncorrected *p*–distance among the *Hemiphyllodactylus* species studied based on mitochondrial ND2 gene fragments.

Species name (*n*)	1	2	3	4	5	6	7	8	9	10	11	12	13	14
1. *H.longlingensis* (4)	–													
2. *H.khlonglanensis* (4)	25.0	–												
3. *H.flaviventris* (5)	41.7	34.9	–											
4. *H.arakuentris* (1)	35.4	35.2	33.1	–										
5. *H.aurantiacus* (2)	37.3	33.9	30.6	26.9	–									
6. *H.jnana* (4)	36.6	35.1	30.1	20.4	17.1	–								
7. *H.kolliensis* (2)	34.1	34.5	30.4	21.8	16.5	10.0	–							
8. *H.kyaiktiyoensis* (4)	27.3	20.3	36.3	41.3	39.2	35.0	36.7	–						
9. *H.pinlaungensis* (3)	28.7	23.1	44.4	44.1	38.1	36.6	37.7	13.1	–					
10. *H.zwegabinensis* (1)	25.6	23.1	39.9	42.5	37.9	37.1	36.0	17.7	21.6	–				
11. *H.zalonicus* (1)	16.1	17.8	40.2	29.3	35.0	32.8	32.7	22.0	25.2	25.7	–			
12. *H.zhutangxiangensis* (5)	21.9	19.2	38.8	30.2	33.4	31.0	28.2	24.9	26.4	26.9	16.6	–		
13. H.changningensis (8)	23.3	23.0	38.5	35.1	36.3	35.4	34.4	25.8	28.3	27.0	14.5	22.2	–	
14. *H.gengmaensis* sp. nov. (5)	19.8	22.7	37.3	32.2	31.2	32.2	34.5	24.5	26.3	24.7	11.5	19.1	9.7	0.3

### ﻿Taxonomic account

#### ﻿ Hemiphyllodactylus gengmaensis sp. nov.

Taxon classificationAnimaliaSquamataGekkonidae

FBAA7F7D-926F-5310-AD3B-93C179F89C5B

https://zoobank.org/16760DC6-5331-4742-A5FE-FD3F6E42DAC8

Figs 4 , 5

##### Material.

***Holotype*.** 2014002302, adult female, collected by Hong Hui on 15 May 2014 from Banxing Village, Gengma Dai and Wa Autonomous County, Yunnan, China (23.517°N, 98.925°E, at an elevation of 664 m).

**Figure 4.**
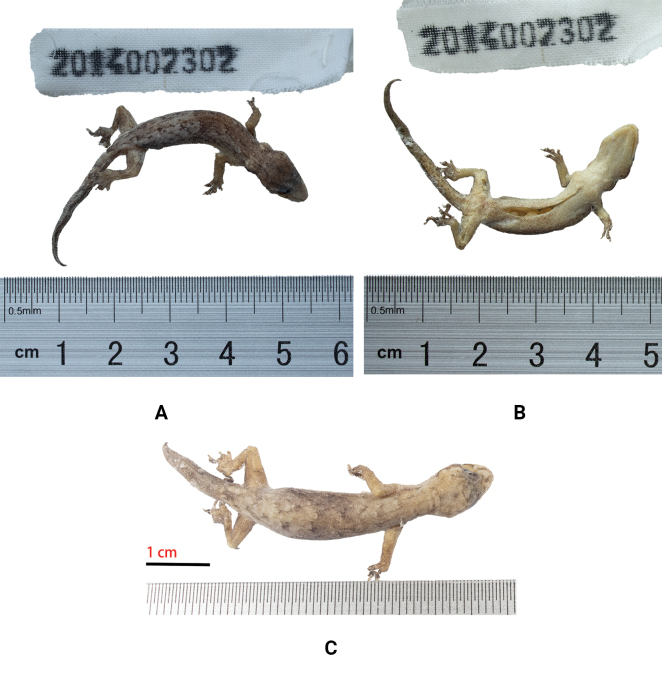
*Hemiphyllodactylusgengmaensis* sp. nov. **A** holotype, 2014002302, dorsal view in alcohol **B** holotype, 2014002302, ventral view in alcohol **C** paratype, 2014002301, dorsal view in alcohol.

***Paratypes*.** 2014002297, 2014002298, 2014002299, adult females, 2014002300, 20140022301, adult males, collected at the same locality as the holotype on 15 May 2014.

**Figure 5.**
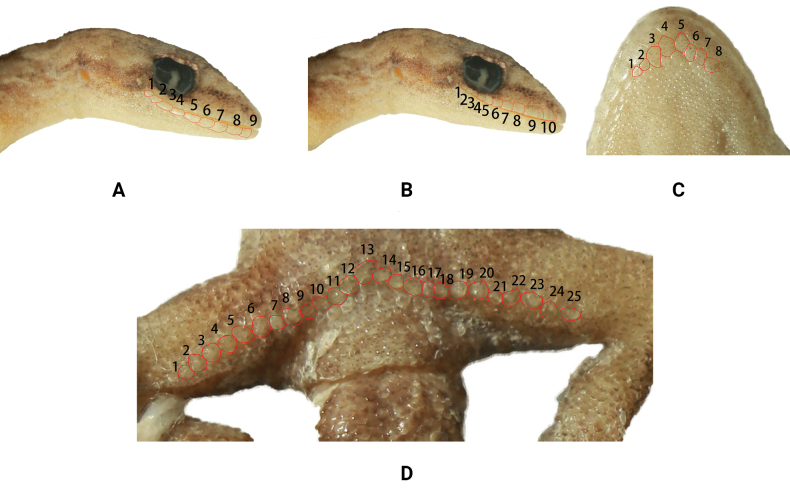
Paratype (2014002301) of *Hemiphyllodactylusgengmaensis* sp. nov. **A, B** lateral views of head, red lines indicate IL and SL, respectively **C** ventral view, red lines indicate chin scales **D** ventral view, red lines indicate femoroprecloacal pores.

##### Diagnosis.

*Hemiphyllodactylusgengmaensis* sp. nov. can be distinguished from its congeners by the combination of the following characters: maximum SVL of 43.24 mm; 8–9 chin scales; enlarged postmentals; 6 circumnasal scales; 2–3 internasal scales; 9–11 supralabial scales; 8–9 infralabial scales; 11–18 dorsal scales; 8–10 ventral scales; a manual lamellar formula of 5–5–5–4 or 5–6–5–4 and a pedal lamellar formula of 5–5–6–5; 20–25 precloacal and femoral pore-bearing scales contiguous in males. dark postorbital stripes or striping on body; dark dorsal transverse blotches; and a brown postsacral mark bearing anteriorly projecting arms.

##### Description of holotype.

Adult female, SVL 38.52 mm; head triangular in dorsal profile, depressed, distinct from neck (HL 10.80 mm; HW 7.36 mm); lores flat; snout short (SnEye 3.94 mm; SnEye/HL 36%), narrow (SnW 1.78 mm; SnW/HW 24%); eye large (ED 2.12 mm; ED/HL 20%); rostral scale wider than high, bordered posteriorly by two large supranasals and three internasals (IS); nares bordered anteriorly by rostral scale, ventrally by first and second supralabial scale, dorsally by supranasal scale, posteriorly by three postnasals; supralabials square, 10/9 (left/right), tapering from rostral to a point in line with posterior margin of orbit (SL); infralabials square, 9/9 (left/right), tapering from mental to a point in line with posterior margin of orbit (IL); scales on head small, rounded, largest on rostrum; mental triangular, eight chin scales touching internal edges of infralabials from juncture of the second and the third on left and right and mental scale (Chin); scales in gular region rounded, non-overlapping, becoming larger and more ovoid on venter. Robust body type and small, (TrunkL/SVL 45%), dorsoventrally compressed; dorsal body scales small, granular, 18 dorsal scales at midbody contained within one eye diameter; ventral body scales smooth and flat, much larger than dorsal scales, subimbricate, 10 ventral scales at midbody contained within one eye diameter; granular scales on the limbs; finger I is vestigial, clawless, and with rectangular subdigital lamellae, while fingers II–V are well developed; the proximal subdigital lamellae are undivided and rectangular, while the distal subdigital lamellae are divided, angular, U-shaped, except for the terminal lamellae, which are rounded and undivided; the forefoot and hindfoot digital formulae are unidentifiable; white cloacal spur present, one on each side. Tail length (TL/SVL = 73%), with dorsal scales on the tail larger than those on the body and head, but smaller than the subcaudals. The ventral scales are large and flat.

##### Coloration in ethanol.

The dorsal surface of head and body is light brown; dark brown stripes extend from the posterior corner of the eye socket to the neck; the back is covered with irregular, dark-brown stripes that interconnect to form a net-like pattern; the dorsal surfaces of the limbs are brown, with irregular, dark-brown stripes; the tail is brown, with several dark-brown, transverse stripes; the regenerated tail is gray and without transverse stripes; the ventral surfaces of the head and limbs are cream-grey.

##### Variation.

Variation of mensural and meristic data are presented in Table 3. Dark dorsal transverse blotches on the body of this species are relatively small, with those of two specimens (2014002302, 2014002300) being indistinct and fragmented. Furthermore, females are slightly larger than males. The postsacral mark, bearing anteriorly projecting arms, of one individual (2014002300) is indistinct, possibly due to prolonged preservation.

**Table 3.** Mensural (in mm), meristic, color pattern, and proportions of the type series of *Hemiphyllodactylusgengmaensis* sp. nov. (–) = data unavailable. (*) = regenerated tail.

Character	Holotype	Paratypes				
	2014002302	2014002300	2014002299	2014002297	2014002298	2014002301
Sex	Female	Male	Female	Female	Female	Male
SVL	38.52	35.24	36.5	40.86	43.24	39.45
TL	28.22	–	9.18*	–	–	15.34*
TrunkL	17.46	17.54	17.6	21.6	23.22	18.82
HL	10.80	10.80	10.24	11.24	11.14	11.24
HW	7.36	6.94	6.88	7.86	8.2	7.74
ED	2.12	2.56	2.16	2.62	2.6	2.34
SnEye	3.94	3.88	4.02	4.5	4.52	4.3
NarEye	2.78	2.74	2.96	3.04	3.32	2.72
SnW	1.78	1.78	1.6	1.84	2.72	1.7
TrunkL/SVL	0.45	0.50	0.48	0.53	0.54	0.48
HL/SVL	0.28	0.31	0.28	0.28	0.26	0.28
HW/SVL	0.19	0.20	0.19	0.19	0.19	0.20
HW/HL	0.68	0.64	0.67	0.70	0.74	0.69
SnEye/HL	0.36	0.36	0.39	0.40	0.41	0.38
NarEye/HL	0.26	0.25	0.29	0.27	0.30	0.24
ED/HL	0.20	0.24	0.21	0.23	0.23	0.21
SnW/HL	0.16	0.16	0.16	0.16	0.24	0.15
ED/NarEye	0.76	0.93	0.73	0.86	0.78	0.86
Snw/HW	0.24	0.26	0.23	0.23	0.33	0.22
Chin	8	9	8	9	9	8
CN	6	6	6	6	6	6
IS	3	2	2	3	3	2
SL (left/right)	10/9	10/10	10/11	10/9	10/9	10/10
IL (left/right)	9/9	9/9	8/8	9/9	9/9	9/9
VS	10	9	9	8	8	9
DS	18	14	11	15	15	16
Lamellar formulae hands II–V (left)	–	–	–	–	5–5–5–4	–
Lamellar formulae hands II–V (right)	–	–	–	–	5–6–5–4	–
Lamellar formulae foot II–V (left)	–	–	–	–	5–5–6–5	–
Lamellar formulae foot II–V (right)	–	–	–	–	5–5–6–5	–
SL1F	–	–	–	–	5	–
SL1T	–	–	–	–	6	–
Precloacal and femoral pore series separate (1) or continuous (0)	–	0	–	–	–	0
Total femoroprecloacal pores	0	20	0	0	0	25
CloacS on each side	1	1	1	1	2	1
Subcaudals enlarged, plate–like	No	No	No	No	No	No
Dark postorbital stripe	Yes	Yes	Yes	Yes	Yes	Yes
Dorsolateral light–colored spots on trunk	No	No	No	No	No	No
Dark dorsolateral stripe on trunk	No	No	No	No	No	No
Dark ventrolateral stripe on trunk	No	No	No	No	No	No
Dark dorsal transverse blotches	Indistinct	Indistinct	Yes	Yes	Yes	Yes
Dark reticulate pattern on dorsum	Indistinct	Yes	Yes	Indistinct	Yes	Yes
Postsacral marking anteriorly projecting arms	Yes	indistinct	Yes	Yes	Yes	Yes

##### Distribution.

This species is currently known to be distributed at the type locality Banxing Village, Gengma Dai and Wa Autonomous County of Yunnan Province in China (Fig. 1).

##### Natural History.

*Hemiphyllodactylusgengmaensis* sp. nov. was found at an elevation of 664 m a.s.l., around 21:00. The specimens were found on a restaurant’s wall, which was rough and with crevices. When illuminated with a flashlight, the animals quickly crawled into the crevices.

##### Etymology.

The scientific name “*gengmaensis*” is derived from its type locality Gengma Dai and Wa Autonomous County in Yunnan province. We suggest Gengma Slender Gecko in English and “耿马半叶趾虎 (Gěng Mǎ Bàn Yè Zhǐ Hǔ)” in Chinese.

##### Comparisons.

We compared the morphology of the new species against its closely related species, specifically species from clade 3, as inferred from the phylogeny we constructed (Table 4). In terms of body proportions, *H.gengmaensis* sp. nov. has a longer head which separates it from *H.longlingensis*, *H.zalonicus*, *H.changningensis*, and *H.zhutangxiangensis* (HL/SVL = 0.26–0.31 versus 0.22–0.24, 0.22–0.23, 0.22–0.25, 0.17–0.20, respectively); greater SnW distance (SnW/HW = 0.22–0.33 versus 0.15–0.18, 0.21, 0.16–0.20, 0.16–0.21, respectively); the new species has a shorter SnEye distance which separates it from *H.longlingensis*, *H.changningensis*, and *H.zhutangxiangensis* (SnEye/HL = 0.36–0.41 versus 0.42–0.45, 0.41–0.49, 0.53–0.60, respectively); shorter head width (HW/HL = 0.64–0.74 versus 0.75–0.80, 0.72–0.80, 1.03–1.13, respectively); it has a shorter NarEye compared to *H.changningensis* and *H.zhutangxiangensis* (NarEye/HL = 0.24–0.30 versus 0.30–0.37, 0.39–0.4, respectively); smaller eyes compared to *H.zalonicus* and *H.zhutangxiangensis* (ED/HL = 0.20–0.24 versus 0.23–0.30, 0.30–0.36, respectively). In terms of scalation, the new species has more CN can be distinguished from *H.longlingensis*, *H.zalonicus*, *H.changningensis*, and *H.zhutangxiangensis* (CN = 6 versus 4–5, 5, 3–4, 5, respectively); more VS compared to *H.longlingensis*, *H.changningensis*, and *H.zhutangxiangensis* (VS = 8–10 versus 6–7, 6–8, 5–7, respectively); the new species has more femoroprecloacal pores which separates it from *H.zalonicus*, *H.changningensis*, and *H.zhutangxiangensis* (20–25 versus 16–20, 19–22, 20–23, respectively). For the lamellar and coloration, the new species differs from *H.longlingensis*, *H.zalonicus*, and *H.changningensis* by having more lamellae on the hand (5–5(6)–5–4 versus 3–4–4–4(3), 3–3(4)–3(4)–3(4) and 3–3(4)–3(4)–3, respectively); differs from *H.zalonicus*, *H.changningensis*, and *H.zhutangxiangensis* by having more lamellae on first fingers (SL1T = 6 versus 4, 3–4, 4–5, respectively). Furthermore, *H.gengmaensis* sp. nov. has dark transverse blotches on the dorsum, which *H.zalonicus* does not have. The new species has a dark, reticulate dorsal pattern, which *H.zalonicus* and *H.zhutangxiangensis* do not have. It has a postsacral marking with anteriorly projecting arms, which is absent in *H.zalonicus* and *H.changningensis*.

**Table 4.** Diagnostic characters separating *Hemiphyllodactylusgengmaensis* sp. nov. from other nominal taxa of *Hemiphyllodactylus* within clade 3 of Agung et al. (2022). (—) = data unavailable. Mensural characters are in mm. Data for *H.zalonicus* and *H.longlingensis* were obtained from Grismer et al. (2020a). Data for *H.changningensis* were obtained from Guo et al. (2015). Data for *H.zhutangxiangensis* were obtained from Agung et al. (2021).

Character	*H.gengmaensis* sp. nov.	*H.longlingensis*	*H.zalonicus*	*H.changningensis*	*H.zhutangxiangensis*
Max SVL	43.24	45.7	37.7	43.8	44.42
*n*	6	–	2	10	13
TrunkL	17.46–23.22	–	18.1–18.9	17.4–22.5	16.1–23.1
HL	10.8–11.24	–	8.4–8.5	8.2–10.1	6.2–7.6
HW	6.88–8.2	–	5.7–5.8	6.1–7.5	6.5–8.2
ED	2.12–2.62	–	2.0–2.6	1.7–2.3	2.1–2.7
SnEye	3.88–4.52	–	3.4–3.5	3.5–4.5	3.4–4.4
NarEye	2.72–3.32	–	2.4–2.7	2.8–3.4	2.6–3.2
SnW	1.6–2.72	–	1.2	1.1–1.4	1.3–1.6
TrunkL/SVL	0.45–0.54	0.47–0.52	0.49–0.50	0.46–0.51	0.48–0.52
HL/SVL	0.26–0.31	0.22–0.24	0.22–0.23	0.22–0.25	0.17–0.20
HW/SVL	0.19–0.2	0.17–0.19	0.15	0.17–0.18	0.18–0.20
HW/HL	0.64–0.74	0.75–0.80	0.68	0.72–0.80	1.03–1.13
SnEye/HL	0.36–0.41	0.42–0.45	0.40–0.42	0.41–0.49	0.53–0.60
NarEye/HL	0.24–0.30	0.29–0.34	0.28–0.31	0.30–0.37	0.39–0.44
ED/HL	0.20–0.24	0.22–0.25	0.23–0.30	0.21–0.25	0.30–0.36
SnW/HL	0.15–0.24	0.12–0.14	0.14	0.12–0.16	0.19–0.22
ED/NarEye	0.73–0.94	0.66–0.82	0.74–1.08	0.61–0.77	0.70–0.91
Snw/HW	0.22–0.33	0.15–0.18	0.21	0.16–0.20	0.16–0.21
Chin	8–9	7–9	8–10	7–8	7–9
CN	6	4–5	5	3–4	5
IS	2–3	1–3	3–4	2–3	2–4
SL	9–11	9–10	10	8–11	8–11
IL	8–9	8–10	8–9	8–10	8–11
VS	8–10	6–7	9–10	6–8	5–7
DS	11–18	10–14	17–18	11–15	11–15
Lamellar formulae hands II–V	5–5(6)–5–4	3–4–4–4(3)	3–3–3–3	3–3(4)–3(4)–3	(3–5)–(4–6)–(4 or 5)–(4 or 5)
		4–4–4(5)–4			
Lamellar formulae feet II–V	5–5–6–5	4–4(5)–4(5)–4	3–4–4–4	3–3–3–3	(4 or 5)–(4 or 5)–(4–6)–(4 or 5)
				3–4–4–4	
SL1F	5	4–5	3	3–4	4–5
SL1T	6	4–6	4	3–4	4–5
Precloacal and femoral pore series separate (1) or continuous (0)	0	0	0	0	0
Total femoroprecloacal pores	20–25	16–27	16–20	19–22	20–23
CloacS on each side	1 or 2	1 or 2	1	1 or 2	1 or 2
Subcaudals enlarged, plate–like	No	No	No	No	No
Dark postorbital stripe	Yes	Yes	Yes	Yes	Yes
Dorsolateral light–colored spots on trunk	No	No	No	No	No
Dark dorsolateral stripe on trunk	No	No	No	No	No
Dark ventrolateral stripe on trunk	No	No	No	No	No
Dark dorsal transverse blotches	Yes or indistinct	Variable	No	Yes	Yes
Dark reticulate pattern on dorsum	Yes or indistinct	Variable	No	Yes	No
Postsacral marking anteriorly projecting arms	Yes or indistinct	Pale colored	Indistinct or not	No	Fork-like, dark colored

## ﻿Discussion

Our research supports the recognition of the *Hemiphyllodactylusgengmaensis* sp. nov. as a new species, belonging to clade 3 of Agung et al. (2021). It is sister taxa to *H.longlingensis*, *H.zalonicus*, *H.changningensis*, and *H.zhutangxiangensis*. Except for *H.zalonicus*, all species of clade 3 occur in China. Considering the 292 km of unexplored area between *H.zalonicus* and its closest distance species (*H.longlingensis*), there may be numerous undescribed species in northern Myanmar and Dehong Dai and Jingpo Autonomous Prefecture, Yunnan Province, China. The 56 km distance between *H.gengmaensis* sp. nov. and *H.changningensis*, along with recent new species discoveries (Grismer et al. 2013; Guo et al. 2015; Che et al. 2020; Agung et al. 2021, 2022), suggests a high diversity of this genus within Yunnan. The border area of Dali Bai Autonomous Prefecture, Pu’er City, and Lincang City in southern Yunnan Province could be a key area for future research.

Our study increases the number of recognized species in the *Hemiphyllodactylus* in China to 15. Apart from *H.typus*, *H.dupanglingensis*, and *H.hongkongensis*, all other species in the genus *Hemiphyllodactylus* are montane species. *Hemiphyllodactylusgengmaensis* sp. nov. was found at an elevation of 664 m a.s.l. The discovery of the new species may represent an intermediate elevation type given by the known ranges, suggesting that species in the *Hemiphyllodactylus* may have a wide distribution range in southern China, spanning elevations from 120 m (Sung et al. 2018) to 2,169 m (unpublished data). Additionally, Grismer et al. (2018b) discovered 12 new gecko species within two weeks in a single study of karsts in Myanmar, with similar climatic and habitat conditions likely to exist in southern China. Moreover, some populations previously considered as *H.yunnanensis* in China have also been described as new species (Deng et al. 1998; Shi et al. 2011; Che et al. 2020; Zhang B et al. 2020). Therefore, it is possible that there are still numerous undiscovered cryptic species in southern China.

## Supplementary Material

XML Treatment for Hemiphyllodactylus gengmaensis

## ﻿Additional information

### Author ORCIDs

Hongxin Zhou https://orcid.org/0009-0004-9801-8811

Shuo Liu https://orcid.org/0000-0001-7825-3006

### Data availability

All of the data that support the findings of this study are available in the main text.
